# Silent Danger: Coexistence of Late‐Onset External Iliac Artery Pseudoaneurysm and Deep Vein Thrombosis

**DOI:** 10.1002/ccd.31715

**Published:** 2025-06-23

**Authors:** Sefa Tatar, Oznur Keskin, Abdullah Enes Atas, Abdullah Icli

**Affiliations:** ^1^ Department of Cardiology, Meram Faculty of Medicine Necmettin Erbakan University Konya Turkey; ^2^ Department of Radiology, Meram Faculty of Medicine Necmettin Erbakan University Konya Turkey

**Keywords:** complication, coronary angiography, deep vein thrombosis, endovascular stenting, pseudoaneurysm

## Abstract

Pseudoaneurysm (PA) is a well‐known vascular complication following percutaneous interventions, typically occurring in the early post‐procedural period. However, delayed‐onset PA associated with deep vein thrombosis (DVT) is an uncommon presentation. This case highlights a late external iliac artery (EIA) PA diagnosed in a patient who initially underwent coronary angiography. A 65‐year‐old male underwent a routine coronary angiography via the femoral artery and was discharged without complications. Twenty days later, he presented with unilateral lower limb swelling and pain. Doppler ultrasonography revealed extensive external iliac DVT, and contrast‐enhanced computed tomography (CT) angiography confirmed a PA of the EIA. Endovascular stent graft placement was performed successfully. The patient was followed for 1 week, during which the limb swelling and pain resolved completely, and he was discharged without complications. This case underscores the importance of considering delayed PA formation in patients with unexplained limb swelling after arterial access procedures. Early diagnosis and intervention are crucial in preventing complications and ensuring favorable outcomes.

## 1 Introduction

Pseudoaneurysm formation is a recognized complication following arterial catheterization procedures, with a typical onset occurring within days after the intervention. However, delayed‐onset pseudoaneurysms, especially those complicated by deep vein thrombosis (DVT), are relatively rare. EIA pseudoaneurysms arise from arterial wall defects leading to blood collection outside the arterial lumen, which is confined by surrounding tissues. If untreated, pseudoaneurysms may enlarge, rupture, or cause significant morbidity. DVT, a frequent complication of vascular interventions, is usually associated with venous stasis, hypercoagulability, or endothelial injury. However, in some cases, a pseudoaneurysm can contribute to venous compression and stasis, increasing the risk of thrombosis. In this report, we present a case of delayed EIA pseudoaneurysm formation associated with ipsilateral lower limb DVT following a routine coronary angiography. This rare presentation highlights the need for vigilance in evaluating post‐procedural limb swelling and underscores the role of endovascular techniques in management.

## 2 Case Presentation

A 56‐year‐old male with no known systemic disease history underwent coronary angiography via the right femoral artery for unstable angina pectoris. A 6 F femoral sheath was placed, and fractional flow reserve (FFR) assessment of the left anterior descending (LAD) artery showed a critical stenosis, leading to the implantation of a 4.0 × 24 mm drug‐eluting stent. Post‐procedurally, aspirin 100 mg daily and ticagrelor 90 mg twice daily were initiated. Three hours after the procedure, the femoral sheath was removed, and hemostasis was achieved with manual compression. Mobilization was performed 9 h later without any evidence of a hematoma or bruit. The patient's hemoglobin level was 13.6 g/dL on the first post‐procedural day, with no significant drop, and he was discharged without complications.

**Figure 1:**
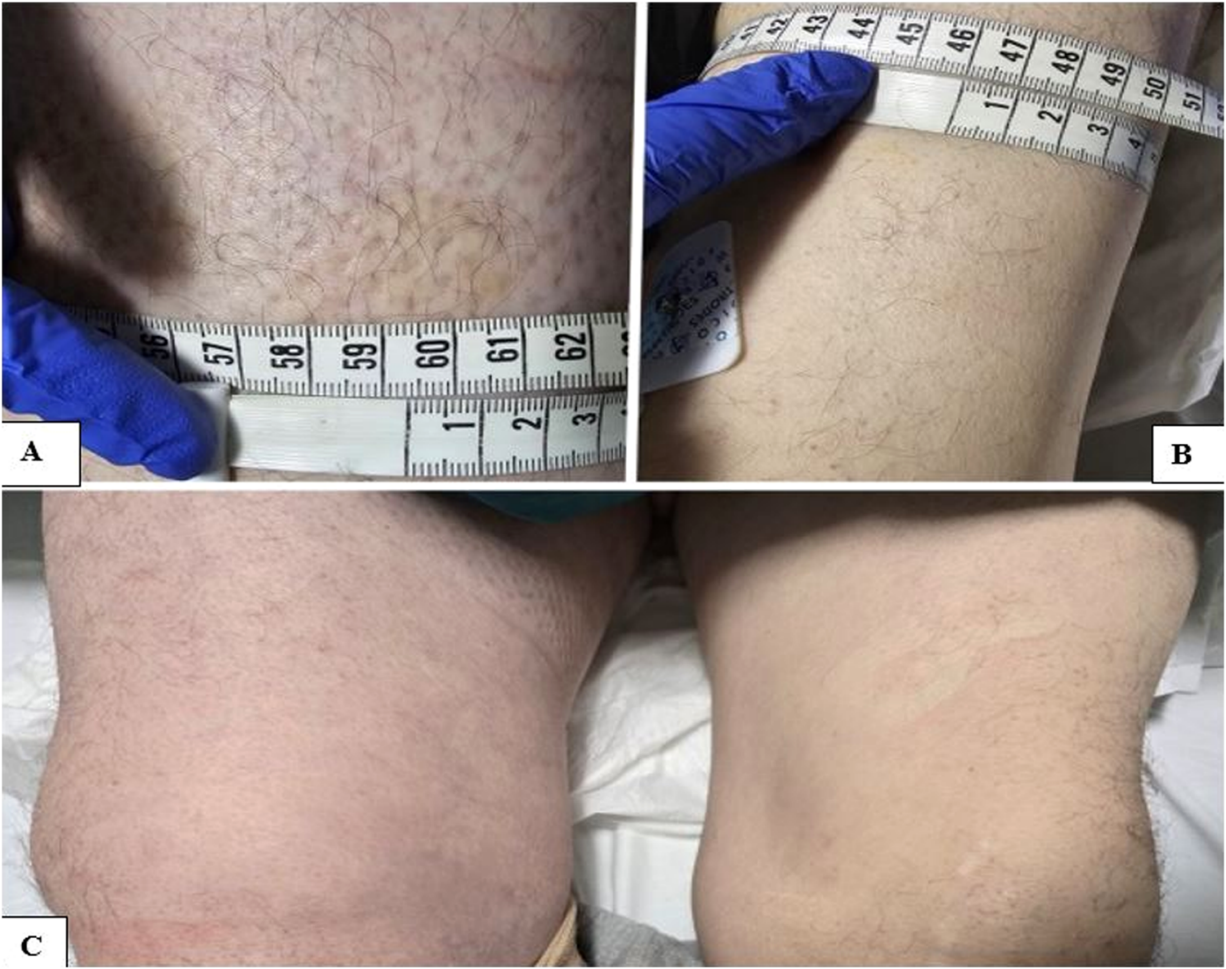
Photographs of the patient at the time of first admission with leg swelling. In picture (A) the diameter of the right leg is seen to be approximately 60 cm, and in picture (B) the diameter of the left leg is seen to be approximately 46 cm. In picture (C) when both legs are examined with inspection, it is seen that there is a difference in diameter from each other. [Color figure can be viewed at wileyonlinelibrary.com]

**Figure 2:**
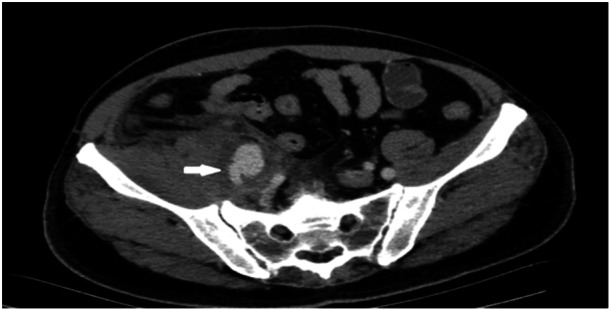
Computerized tomographic angiography performed on the patient shows deep vein thrombosis due to pseudoaneurysm compression in the right femoral vein.

**Figure 3:**
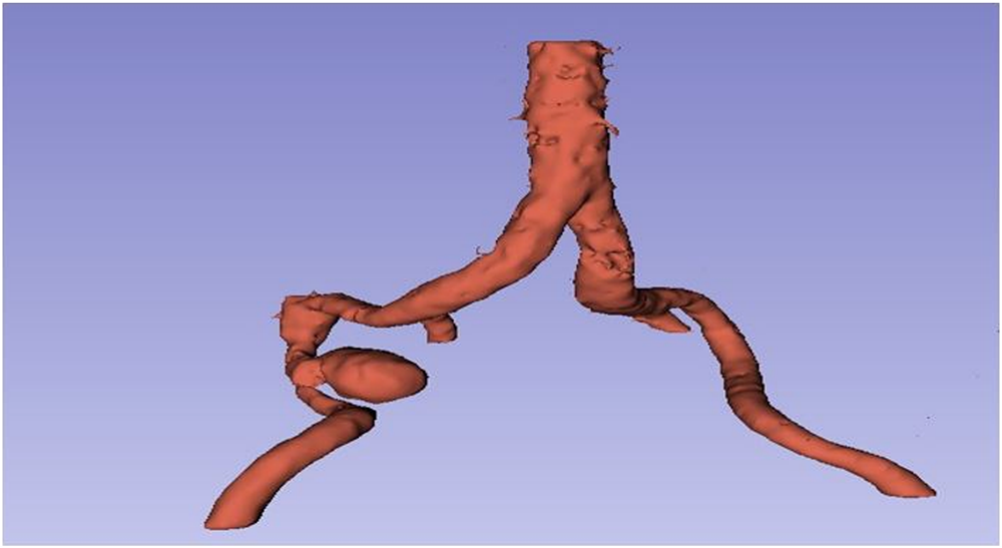
3D computed tomography image of a pseudoaneurysm in the right external iliac artery. [Color figure can be viewed at wileyonlinelibrary.com]

Twenty days later, the patient presented to the emergency department with extensive swelling, pain, and discoloration of the right leg (Figure 1). On physical examination, he had tachycardia and hypotension, with a 12 cm circumference difference between both legs. CT angiography revealed a 3.5 × 4 cm pseudoaneurysm in the right external iliac artery, causing compression on the external iliac vein and surrounded by edema (Figures 2 and 3). The hemoglobin level was noted to be 10.6 g/dL. Doppler ultrasonography showed subacute thrombus formation in the right common femoral vein with slow venous flow. Transthoracic echocardiography revealed normal right heart dimensions and a pulmonary artery pressure of 25 mmHg, without findings suggestive of pulmonary embolism.

**Figure 4:**
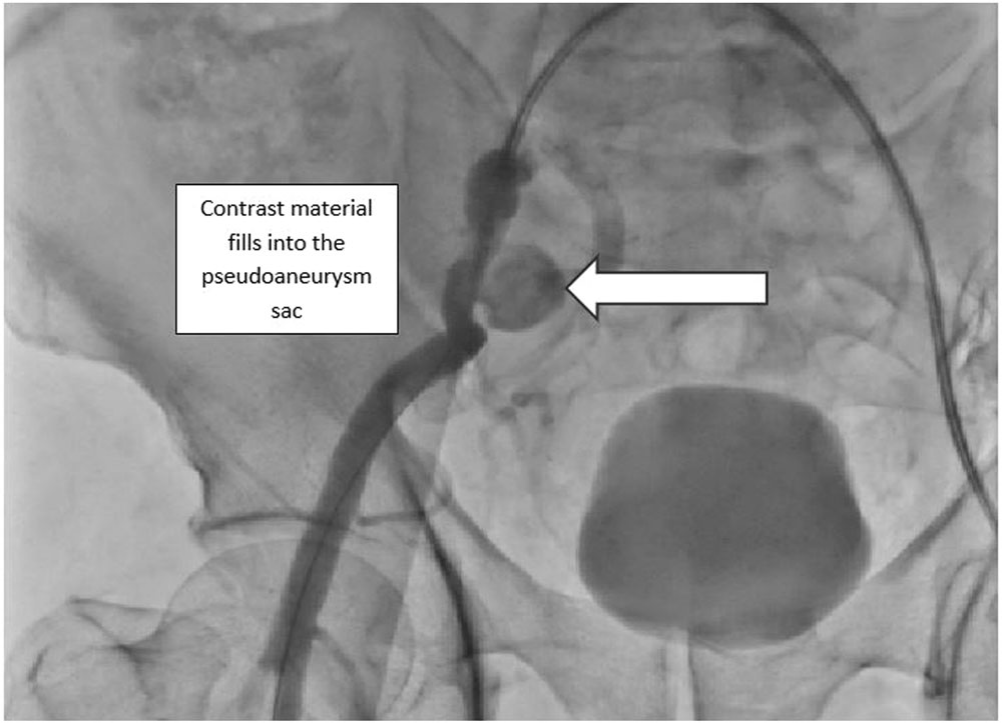
The patient's right external iliac artery was imaged by performing an intervention from the left femoral artery. Angiographic image of the pseudoaneurysm in the right external iliac artery.

**Figure 5:**
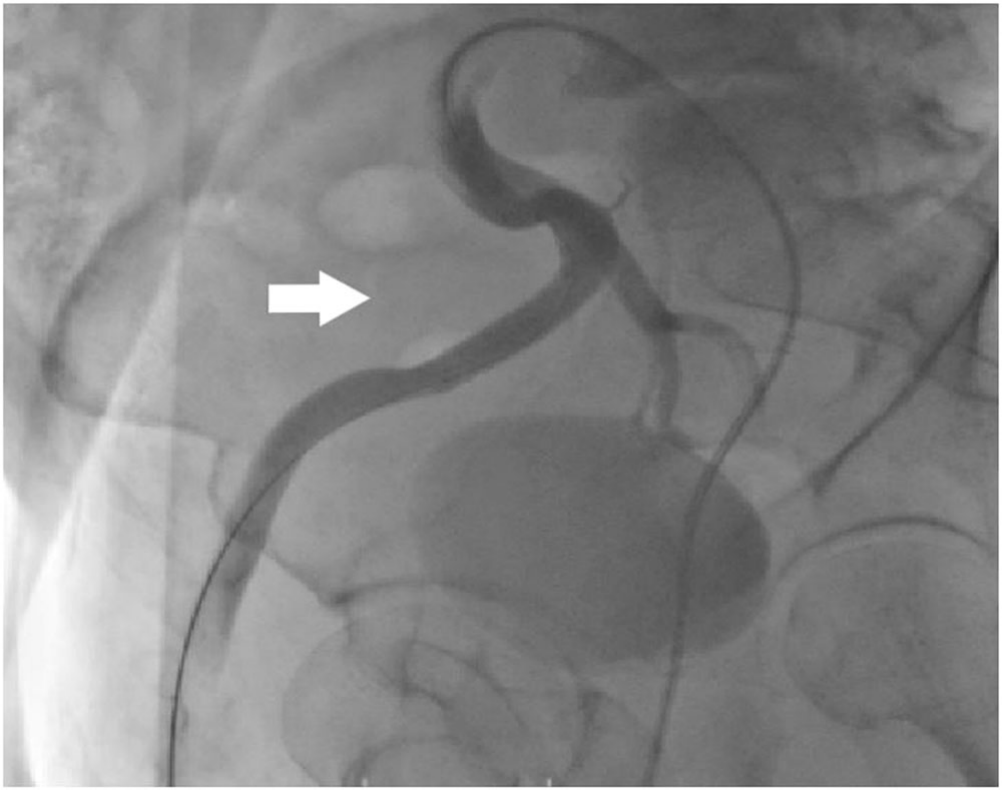
After the greft stent was placed in the right external iliac artery, the pseudoaneurysm sac disappeared and there was no contrast medium filling.

**Figure 6:**
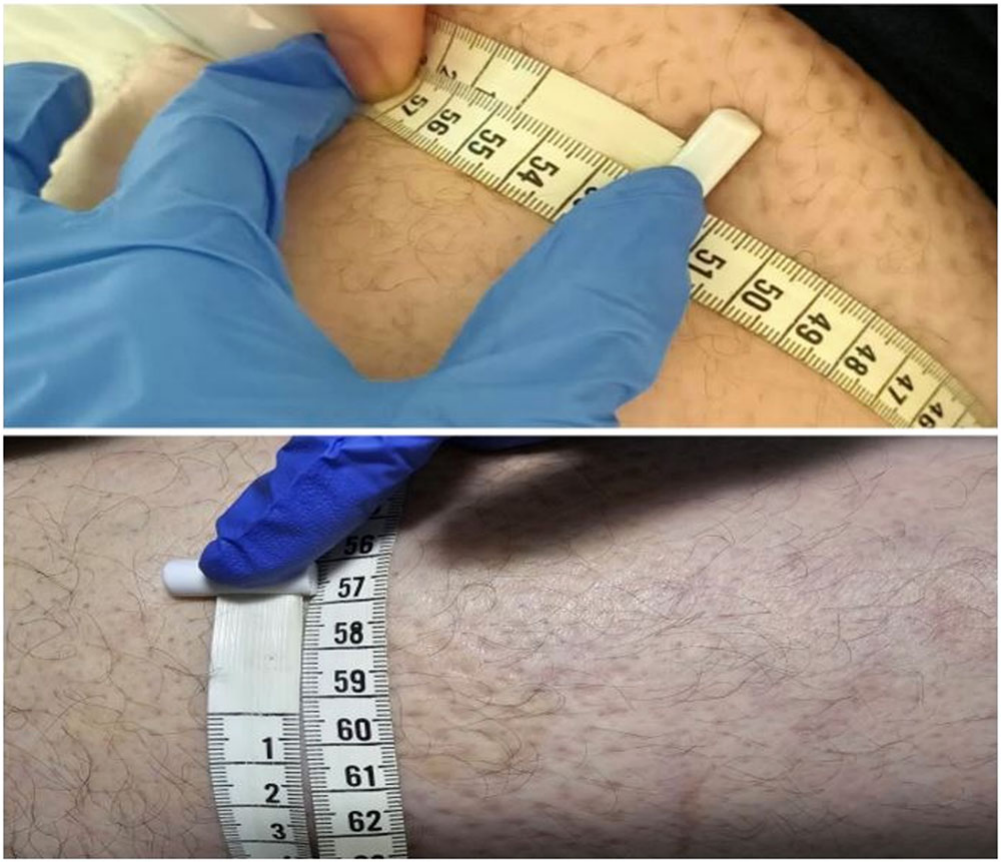
A picture taken 2 days before discharge. The difference in diameter between the patient's legs has been seen to have decreased dramatically. [Color figure can be viewed at wileyonlinelibrary.com]

Consultation with cardiovascular surgery resulted in the decision to perform a percutaneous intervention. Access was obtained via the left femoral artery, and digital subtraction angiography confirmed the pseudoaneurysm. Due to the risk of rupture, a 10 × 37 mm covered stent was deployed to exclude the pseudoaneurysm. The pseudoaneurysm flow was successfully obliterated (Figures 4 and 5). The patient was started on enoxaparin 6000 IU twice daily in addition to dual antiplatelet therapy. By the third post‐procedural day, the leg circumference had decreased by approximately 4 cm (Figure 6). The patient was mobilized and discharged on the fifth post‐procedural day with no complications.

## 3 Discussion

EIA pseudoaneurysms are significant vascular complications following percutaneous interventions. While most cases present in the early post‐procedural period, delayed PA development remains a rare but challenging entity. This case describes a delayed EIA pseudoaneurysm associated with DVT, highlighting the need for careful follow‐up even after seemingly uncomplicated procedures.

Pseudoaneurysms develop due to the disruption of the arterial wall, leading to extravasation of blood into surrounding tissues and subsequent encapsulation by a fibrous sac. Risk factors include inadequate hemostasis, large‐bore catheter use, obesity, anticoagulant therapy, and repeated punctures. Studies have reported an incidence of pseudoaneurysm formation following arterial catheterization ranging from 0.2% to 0.3% [1]. However, cases presenting beyond the immediate post‐procedural period, as in this patient, are exceptionally rare.

The association between pseudoaneurysms and DVT is well‐documented but uncommon. An expanding pseudoaneurysm can compress adjacent venous structures, leading to venous stasis and thrombosis [2]. This mechanism was evident in our case, where the pseudoaneurysm compressed the external iliac vein, contributing to DVT formation. Delayed complications may occur due to inexperience in catheter manipulations and wire use, especially in operators who are new to interventional procedures.

The primary imaging modality for diagnosing pseudoaneurysms is Doppler ultrasonography, which provides detailed information on the lesion's size, flow characteristics, and associated thrombotic changes [3]. Contrast‐enhanced CT angiography further aids in delineating anatomical relationships and assessing potential complications [4].

Treatment options include ultrasound‐guided compression, thrombin injection, endovascular stent graft placement, and open surgical repair. The choice of therapy depends on factors such as pseudoaneurysm size, location, and associated complications. Literature suggests that thrombin injection is highly effective for small pseudoaneurysms, while endovascular stenting is preferred for larger or symptomatic cases [5, 6]. In this case, the pseudoaneurysm's size and the presence of DVT necessitated covered stent deployment.

Endovascular techniques have gained widespread acceptance due to their minimally invasive nature, shorter recovery times, and favorable long‐term outcomes [7]. However, complications such as endoleaks, restenosis, or graft thrombosis must be considered in follow‐up evaluations [8, 9]. Preventative measures, including adequate hemostasis, optimal puncture site selection, and careful patient monitoring, can significantly reduce pseudoaneurysm formation [10, 11].

## 4 Conclusion

Delayed EIA pseudoaneurysms remain a rare but potentially serious vascular complication. This case emphasizes the importance of recognizing and managing late‐onset pseudoaneurysms, particularly those associated with DVT. A multidisciplinary approach, early diagnosis, and appropriate intervention are essential to prevent life‐threatening complications and improve patient outcomes.

## Conflicts of Interest

The authors declare no conflicts of interest.

## Data Availability

The original contributions presented in the study are included in the article, further inquiries can be directed to the corresponding author.
